# Assessment and factors affecting quality of life among patients with Wilson’s disease

**DOI:** 10.1038/s41598-024-59377-w

**Published:** 2024-04-15

**Authors:** Tingting Zhan, Yuxiang Guan, Caijie Sun, Lili Wang, Yan Wang, Xiang Li

**Affiliations:** 1 Department of Brain Center, The First Affiliated Hospital of Anhui University of Chinese Medicine, Hefei, 230031 China; 2 School of Nursing, Anhui University of Traditional Chinese Medicine, Hefei, 230031 China

**Keywords:** Wilson’s disease, Quality of life, Depression, Anxiety, Copper deposits, Diseases, Risk factors

## Abstract

Wilson’s disease is caused by abnormal copper metabolism resulting in deposition in various organs, including the brain, liver, and cornea, thus disrupting organ function. It is characterized by encephalopathy, extrapyramidal symptoms, progressive liver failure, and copper ring deposition in the cornea. Management of this disease should include quality of life maintenance; however, relevant studies on this topic are lacking. This study aimed to assess the factors affecting the quality of life (QoL) of patients with Wilson’s disease. A cross-sectional survey using convenience sampling was conducted between July 2020 and March 2021 at the hospital. Data on patient characteristics, 36-item Short-Form General Health Survey, Uniform Wilson Disease Rating Scale, and Hamilton Depression Rating Scale scores were collected. Associations among quality of life depression, anxiety, and Wilson’s disease progression were examined using Pearson correlation analysis. Factors affecting the quality of life of patients, including depression, anxiety, liver function, clinical symptoms, diet, liver function, brain magnetic resonance imaging (MRI) findings, disease duration, Barthel Index, and Morse scores were examined using multivariate linear regression analysis. This study included 134 patients with Wilson’s disease whose mean age was 29.12 ± 8.59 years. The mean QoL score in the patient group was 71.38 ± 9.55 points and was negatively correlated with anxiety (*r* = − 0.883, *P* = 0.000), depression (*r* = − 0.852 *P* = 0.000), and clinical symptoms (*r* = − 0.542, *P* = 0.000) scores. Anxiety, depression, and clinical symptoms severity are vital factors for the QoL of patients with Wilson’s disease. The study provides foundational evidence to design novel interventions, including symptom management, diet, and self-care ability, which can help in improving the quality of life in patients with Wilson’s disease and decreasing the burden associated with this disease.

## Introduction

Wilson’s disease, also known as hepatolenticular degeneration, is a rare recessive autosomal disorder of the nervous system, characterized by chronic and progressive copper ion metabolism disorder^1^. The prevalence of Wilson’s disease is estimated between 1/100.000 to 1/3 million people, with 1/90 people suspected carriers^2^. The incidence rate of Wilson’s disease is high in Italy, Bulgaria, Israel, Romania, and other European countries. In Asia, the incidence rate has increased in Korea and Japan and increased even further in China^3^. With a large population denominator, China has a high burden of diseases. Therefore, understanding the burden of Wilson’s disease is paramount.

Abnormal copper metabolism in patients with Wilson’s disease results in a large amount of free copper ions accumulating over time in the liver, brain, cornea, and other tissues and organs. Above a certain threshold, the function of these organs becomes abnormal^4^. Individual differences determine copper metabolism, including absorption speed, organ distribution, and accumulation rate. The clinical manifestations of this disease are complex and diverse, and the initial symptoms differ. Progressive liver injury, extrapyramidal symptoms, mental abnormalities, kidney injury, and other symptoms are common^5^. Extra vertebral system symptoms tend to be prominent and include tremors, dysarthria, dystonia, and extensive nervous system damage. Liver damage is the most common manifestation of Wilson’s disease and may present with elevated levels of serum transaminases (asymptomatic), fatty liver, progressive liver fibrosis, irreversible liver cirrhosis, or rapidly progressing liver failure^6,7^. Medical progress has improved the management of Wilson’s disease resulting in extending patients’ life expectancy and enabling activities of daily living, making it a chronic disease. Research in chronic disease has examined factors affecting the quality of life (QoL) of affected patients^8^. The QoL refers to the overall well-being and accounts for physical and psychological factors, including behavioral patterns, sleep quality, energy levels, spiritual health, and general life satisfaction. The QoL is an indicator of treatment effectiveness in chronic disease^9^, specifically where treatment methods are for long term and are associated with side effects^10,11^. However, with increased availability of medical treatment and clinical management, the QoL of patients with Wilson’s disease remains unclear. Therefore, this study investigated factors associated with the QoL of patients with Wilson’s disease in China, providing foundational evidence to develop contextual interventions.

## Methods

This cross-sectional survey used convenience sampling, and 140 patients with Wilson’s disease hospitalized between July 2020 and March 2021 were included. Patients were eligible for this study if they: provided signed informed consent; were aged ≥ 18 years; and presented with normal behavior. Patients who refused to participate, were aged < 18 years, presented with abnormal behavior, lacked the capacity to communicate, and presented with poor adherence were excluded from the analysis.

### Data collection

The research team designed the questionnaire to reflect the study aims, that was further modified by two chief physicians and two deputy chief nurses to reflect the clinical context. Data on variables such as age, sex, diet, stool quality, urine output, pulse, dialectical typing, liver function, urine composition, copper oxidase level, ceruloplasm in levels, corneal K-F ring presence, and Barthel Index, Morse, and Braden scores was collected.

The Short-Form General Health Survey (SF-36) assesses the QoL, and it was translated into Chinese language^12^. The SF-36 includes 36 items that assess health-related QoL across eight dimensions: physical function, role-physical, pain, general health, vitality, social function, role-emotional, and mental health, each of which contain 2–10 entries. The validity and reliability of this scale are satisfactory, with a Cronbach’s α of 0.869^13^ and a total score ranges between 0 and 100 points, where higher scores represent better QoL.

The Uniform Wilson’s disease Rating Scale (UWDRS) was developed^14^ to assess severity of clinical symptoms and drug efficacy in patients with Wilson’s disease. This scale is a recognized tool for evaluating severity, progression, prognosis, treatment efficacy, and outcomes in Wilson’s disease in China^15^. It includes 55 items across the 27 neurological, nine liver, and nine psychiatric domains. The scale uses a 5-point scoring method, each item is scored on a 4-point scale, with a total score of 320 points and higher scores represent greater symptom severity.

The Hamilton Depression Rating Scale^16^ includes 14 items scored on a 4-point scale, wherein scores of > 35, 20–34, 8–20, and < 8 points represent severe, moderate, mild, and absence of depression, respectively. The Hamilton Anxiety Scale^17^ includes 14 items that assess physical and mental anxiety, and scores of 14–21, 22–29, and ≥ 30 points represent mild, moderate, and severe anxiety, respectively. The investigators underwent training and were instructed to use unified language in this study. Eligible patients with Wilson’s disease were informed about the purpose of this study before they provided their consent to participate and undergo assessments. The patients’ completed the questionnaires, and those who could not complete the questionnaires independently were interviewed by the investigators who recorded their answers. The questionnaires were verified carefully. In cases of omissions, respondents were asked to provide missing answers. Each survey lasted for 30–35 min. A total of 140 questionnaires were distributed in this study, and the response rate was 95.7%.

### Statistical analyses

Data analyses were carried out using the Statistical Package for Social Sciences (SPSS) version 22.0 (IBM SPSS Statistics; Chicago). Normally distributed data was presented as means with standard deviations. Univariate analysis of normally distributed data was performed with the independent t-test; multivariate analysis of data with uniform variance was performed using analysis of variance. The QoL score was the dependent variable in multivariable regression. Factors associated with the QoL scores in univariate analysis were included in multivariate analysis. Pearson correlation analysis was used to examine associations of the QoL scores with anxiety, depression, and Wilson’s disease progression. The *P*-values of < 0.05 indicated statistical significance. The number of variables in this study was 15. The required sample size estimation was based on the principle that sample should be 5–10 times greater than the number of variables of interest. The sample size accounted for a 20% drop-out rate and yielded 90–180 participants^18^. The study was conducted in accordance with the Declaration of Helsinki, the Ethics Committee of the First Affiliated Hospital of Anhui University of Traditional Chinese Medicine approved this study (approval number 2021AH-06). Written informed consent was obtained from all patients participating in the study.

## Results

This study included 134 patients (82 [61.2%] men), with 93 and 41 patients in the age groups of 18–35 years and 36–55 years, respectively. Disease durations were ≤ 5, 6–10, and > 10 years in 27 (20.1%), 63 (47%), and 44 (32.8%) patients, respectively (Table S1). The average QoL score was 71.38 ± 9.55 points, and dimension scores are presented in Table S2.

The QoL score was negatively correlated with depression, anxiety, and clinical symptom scores (*P* < 0.05) (Table S3). Disease duration, diet, liver function, Morse score^19^, Barthel Index^20^, and brain magnetic resonance imaging (MRI) findings were associated with QoL scores (*P* < 0.05). Specifically, sex, age, Traditional Chinese Medicine syndrome type, urine output, copper oxidase levels, ceruloplasmin levels, or 24-h urinary copper content were not associated with the QoL in this context (*P* < 0.05) (Table S1). The results of multiple linear regression analysis (Table S4) show that the anxiety, depression, clinical symptoms, diet, liver function, brain MRI findings, disease duration, Barthel Index, and Morse scores were significantly associated with the QoL in patients with Wilson’s disease. Anxiety and depression impacted the QoL and were associated with standardized regression coefficients of − 0.478 and − 2.87, respectively (Tables S4 and S5).

## Discussion

Research on QoL is multidimensional and aims to capture the impact of disease and treatment on the activities of daily living. The concept captures individual attitudes to life, including cultural perception, values, relationships, goals, expectations, and concerns. Overall, QoL refers to physical and psychological health, independence, social competencies, beliefs, and relationship with the environment^21^, making it a subjective measure. Maintaining QoL is a primary goal of chronic disease management; however, research on QoL with a focus on patients with Wilson’s disease is lacking. In this study, the average SF-36 score was 71.38 ± 9.55 points. This value is consistent with that previously reported, suggesting the generalizability of this finding to patients with Wilson’s disease treated in other contexts^22^. The QoL scores in patients with Wilson’s disease tend to be lower than those in patients with chronic hepatitis B. Wilson’s disease is associated with reduced liver dysfunction and is not contagious; nevertheless, the QoL of patients with Wilson’s disease remains low.

In our study, one-way analysis of variance revealed that disease duration, diet, liver function, risk of falling, self-care ability, and brain MRI findings were associated with the QoL. The QoL declined with disease duration; patients with disease duration > 10 years had an average QoL score of 68.090 ± 9.41 points. In addition, over time, the precipitation of copper ions results in symptoms such as dystonia, limb shaking, and salivation, among others, which severely limit the QoL.

Patients who consume a low-copper diet have a higher QoL than those who consume a normal diet. The low-copper diet restricts the intake of food high in copper such as seafood, animal offal, and soy products. This may be due to reduced access to these delicacies in specific cultures, negatively affecting the QoL. In this study, patients with normal liver function had a better QoL than those with abnormal liver function. Among patients with hepatic Wilson’s disease, 41% had abnormal liver function. Patients with abnormal liver function may experience cirrhosis, affecting physical, psychological, and social functioning. The QoL among patients with Wilson’s disease combined with Morse scores ≥ 45 points, was lower than that reported in those with scores < 45. This suggested that an increased risk of falls reduces QoL. This is a novel finding. Morse score assesses the risk of falls based on history, presence of comorbidities, walking aid use, intravenous fluid need, gait, and mental state, that affect QoL in patients with Wilson’s disease. The risk of falling does affect the QoL in older adults with macular degeneration^23^.

Patients with greater ability for self-care had higher QoL scores. This result is consistent with Orem's theory of self-care, which postulates that self-care affects health, happiness, and the QoL in individuals^24^. In addition, patients with abnormal brain MRI findings had poorer QoL than those with normal brain scan findings. Brain lesions were observed in the lenticular nucleus, midbrain, pons, thalamus, external capsule, internal capsule hindlimb, caudate nucleus head, and cerebellar foot. Copper accumulation in different brain structures results in corresponding clinical symptoms. Abnormal MRI findings are associated with Wilson’s disease severity^25^ and severe clinical symptoms do reduce the QoL.

Patients with Wilson’s disease may present with indifference and anxiety^26^, which may progress to clinical challenges. In this study, the average anxiety and depression scores were 23.28 ± 10.33 and 16.86 ± 7.33 points, respectively, indicating mild symptoms. Anxiety and depression may distort patients’ understanding of their health and disease and may result in loss of patients’ confidence in treatment, patients becoming more passive in interpersonal communication, and reduced social activities, leading to alienation and loss of social support, all of which reduce the QoL, aggravating depression and anxiety. Many patients presenting at clinics have psychological symptoms that require interventions, including medication, to help manage mood disorders and support them in adapting to their diagnosis. This approach may help reduce anxiety and depression and improve confidence in treatment, thus maintaining better QoL.

In this study, the UWDRS assessed the clinical symptoms of patients with Wilson’s disease. The average score was 53.62 ± 19.28 points, and it was negatively associated with the QoL. This result is consistent with that of a previous study^27^, that has shown a negative correlation between clinical severity and physical QoL scores. Other studies reported similar findings^26^. Clinical management should aim to reduce symptom severity and improve QoL scores.

Multivariate linear regression analysis revealed that depression and anxiety are the key factors associated with QoL in patients with Wilson’s disease. Wilson’s disease does affect mental health at any stage of disease. In fact, 70–80% of patients with Wilson’s disease have mental health problems^28^, with anxiety and depression accounting for a considerable proportion of cases^29^. Wilson’s disease restricts patients’ physiological function. Long-term treatment, repeated hospitalizations, and significant treatment costs may trigger depression and anxiety, reducing the QoL. As Wilson’s disease progresses, patients may develop dystonia, dysphagia, walking difficulties, reduced self-care ability, and reduced QoL, all of which may aggravate anxiety and depression. This presentation may coincide with decreased appetite, lifestyle changes, and further QoL reduction. Depression and anxiety have profound effect on the QoL of patients with Wilson’s disease and should have a separate focus in treatment protocols. The Bushen Yanggan decoction was used to relieve the symptoms of anxiety and depression in patients with Wilson’s disease to improve QoL^30^. Comprehensive nursing interventions will help improve the QoL in this context^31^.

This study has limitations. First limitation is the sampling method used. In addition, patients with Wilson’s disease often present with cognitive or behavioral disturbances, that may have affected the reliability of the answers provided by patients. Future studies should include a self-awareness scale to improve data reliability.

## Conclusions

Anxiety, depression, and clinical symptoms severity are vital factors for the QoL of patients with Wilson’s disease. This study examined the QoL of patients with Wilson’s disease in China and determined factors that affect it, thus providing foundational evidence for interventions aimed at improving the QoL in this patient group.

### Supplementary Information

Supplementary Tables.

## Data Availability

The datasets generated and/or analysed during the current study are not publicly available because it contains personal privacy information, but are available from the corresponding author on reasonable request.
