# Supplementary material for: Assessment and factors affecting quality of life among patients with Wilson’s disease
Source: Sci Rep. 2024 Apr 15;14:8636. doi: 10.1038/s41598-024-59377-w (PMC11018809; doi:10.1038/s41598-024-59377-w)
Supplement: Supplementary file 1 — Supplementary Tables. [file 41598_2024_59377_MOESM1_ESM.docx]

| **Variable** |  | **n** | **Composition**  **ratio (%)** | **QoL**  **(mean±SD)** | **t/F** | **P-value** |
| --- | --- | --- | --- | --- | --- | --- |
| Sex | Male | 82 | 61.2 | 71.487±9.081 | 0.163 | 0.18 |
|  | Female | 52 | 38.8 | 71.211±10.341 |  |  |
| Age (years) | 18–35 | 93 | 69.4 | 70.290±9.210 | -2.012 | 0.516 |
|  | 36–55 | 41 | 30.6 | 73.853±9.963 |  |  |
| Disease duration (years) | ≤5 | 27 | 20.1 | 78.370±10.042 | 11.599 | 0 |
|  | 6–10 | 63 | 47 | 70.682±7.914 |  |  |
|  | >10 | 44 | 32.8 | 68.090±9.407 |  |  |
| Diet | Low-copper diet | 118 | 88.1 | 69.178±7.745 | -9.284 | 0.009 |
|  | Light diet | 16 | 11.9 | 87.625±4.645 |  |  |
| Stool | Normal | 121 | 90.3 | 71.570±9.620 | 0.948 | 0.419 |
|  | Constipate | 6 | 4.5 | 74±7.483 |  |  |
|  | Dry knot | 6 | 4.5 | 66±9.919 |  |  |
|  | Loose stools | 1 | 0.7 | 65 |  |  |
| Urinate | Normal | 129 | 96.3 | 71.689±9.301 | 1.838 | 0.163 |
|  | Less | 3 | 2.2 | 63.666±13.051 |  |  |
|  | More | 2 | 1.5 | 63±19.798 |  |  |
| Pulse condition | Slide pulse | 112 | 83.6 | 71.008±8.991 | 0.538 | 0.708 |
|  | String pulse | 11 | 8.2 | 74.636±10.846 |  |  |
|  | Slow pulse | 2 | 1.5 | 73±33.941 |  |  |
|  | Astringent pulse | 4 | 3 | 68.750±8.808 |  |  |
|  | Fine pulse | 5 | 3.7 | 74±10.024 |  |  |
| TCM syndrome type | Damp and heat content | 70 | 52.2 | 71.7±9.665 | 0.263 | 0.901 |
|  | Phlegm and blood stasis | 58 | 43.3 | 71.155±9.772 |  |  |
|  | Liver and kidney deficiency | 3 | 2.2 | 66.667±8.621 |  |  |
|  | Gas and blood deficiency | 1 | 0.7 | 70 |  |  |
|  | Liver gas stagnation | 2 | 1.5 | 74.5±0.707 |  | |
| Liver function | Normal | 15 | 11.2 | 88.066±4.447 | 9.138 | 0.007 |
|  | Abnormal | 119 | 88.8 | 69.277±7.788 |  |  |
| Urine findings | Normal | 101 | 75.4 | 71.297±9.432 | 0.025 | 0.995 |
|  | Red blood cell abnormalities | 13 | 9.7 | 71.769±11.166 |  |  |
|  | White blood cell abnormalities | 13 | 9.7 | 71.230±9.696 |  |  |
|  | Red and white blood cells are abnormal | 7 | 5.2 | 72.142±10.073 |  |  |
| Copper oxidase | ≤0.26 | 104 | 77.6 | 70.528±9.312 | 3.77 | 0.054 |
|  | 0.27–0.65 | 30 | 22.4 | 74.333±9.945 |  |  |
| Ceruloplasmin | ≤0.23 | 38 | 28.4 | 73.157±6.906 |  |  |
|  | 0.24–0.44 | 96 | 71.6 | 70.677±10.364 |  |  |
| Five elements of whole blood | Normal | 46 | 34.3 | 73.5±8.334 | 1.75 | 0.178 |
|  | Copper rise | 33 | 24.6 | 70.121±10.37 |  |  |
|  | Copper reduction | 35 | 41 | 70.363±9.864 |  |  |
| 24-h urine copper output | Normal | 10 | 7.5 | 70.1±6.723 | 0.097 | 0.907 |
|  | Elevated | 123 | 91.8 | 71.479±9.798 |  |  |
|  | Reduced | 1 | 0.7 | 72 |  |  |
| Cranial MRI findings | Normal | 9 | 14.3 | 86.315±5.271 | 9.516 | 0.041 |
|  | Abnormal | 115 | 58.5 | 68.913±7.665 |  |  |
| Corneal K-F ring | Positive | 109 | 81.5 | 71.724±10.005 | 0.141 | 0.87 |
|  | Negative | 25 | 18.7 | 69.880±7.236 |  |  |
| Braden score | 9–12 | 1 | 0.7 | 49 | 2.959 | 0.055 |
|  | 13-18 | 1 | 0.7 | 46 |  |  |
|  | ≥18 | 132 | 98.5 | 71.515±9.415 |  |  |
| Morse score | ≤45 | 12 | 9 | 89.333±4.030 | 8.431 | 0.008 |
|  | >45 | 122 | 91 | 69.614±7.982 |  |  |
| Barthel index score | <60 | 117 | 87.3 | 69.085±7.713 | -9.386 | 0.013 |
|  | ≥60 | 17 | 12.7 | 87.176±4.863 |  |  |

**Table S1.** Demographic and clinical characteristics of patients with Wilson’s disease

| **Item** | **Number of items** | **Score (**mean±SD) |
| --- | --- | --- |
| Physiological function | 10 | 18.91±5.77 |
| Role-physiological function | 4 | 4.54±1.82 |
| Affective function | 4 | 6.44±2.21 |
| Body pain | 2 | 5.79±2.01 |
| Energy | 3 | 9.42±3.24 |
| Mental health | 6 | 12.54±5.74 |
| Social function | 1 | 3.69±1.57 |
| General health | 6 | 10.07±7.04 |
| Total score | 36 | 71.38±9.55 |

**Table S2**. Quality of life dimension scores in patients with Wilson’s disease (n=134)

| Quality of life | | |
| --- | --- | --- |
| **Item** | **Pearson correlation coefficient** | **P-values** |
| HAMA | -0.883 | 0.000 |
| HAMD | -0.852 | 0.000 |
| UWDRS | -0.542 | 0.000 |
| HAMA | -0.883 | 0.000 |

**Table S3**. Correlations between quality of life, depression, anxiety, and clinical symptom scores in patients with Wilson’s disease

HAMD: Hamilton Depression Rating Scale, HAMA: Hamilton Anxiety Scale, UWDRS: Uniform Wilson Disease Rating Scale

| **Variable name** | **Number of items** | **Variable assignment** |
| --- | --- | --- |
| X1 | Anxiety | Actual value |
| X2 | Depression | Actual value |
| X3 | UWDRS | Actual value |
| X4 | Diet | Low-copper diet=1, Light diet=2 |
| X5 | Liver function | Normal=1; Abnormal=2 |
| X6 | Cranial MRI | Normal=1; Abnormal=2 |
| X9 | Morse score | ≦45=1; >45=2 |
| Y | QoL | Actual value |

**Table S4**. Factors associated with the quality of life of patients with Wilson disease

| **Variable** | **B value** | **SE** | **β** | **t** | ***P-value*** |
| --- | --- | --- | --- | --- | --- |
| Constant | 107.993 | 12.800 |  | 8.437 | 0.000 |
| HAMA | -0.442 | 0.059 | -0.478 | -7.504 | 0.000 |
| HAMD | -0.374 | 0.082 | -0.287 | -4.544 | 0.000 |
| UWDRS | -0.047 | 0.02 | -0.094 | -2.350 | 0.02 |
| Diet | -4.599 | 5.566 | -0.157 | -0.826 | 0.04 |
| Liver function | -3.213 | 4.186 | -0.106 | -0.768 | 0.002 |
| Cranial MRI findings | -1.497 | 2.190 | -0.055 | -0.684 | 0.009 |
| Disease duration (years) | -0.026 | 0.492 | -0.002 | -0.054 | 0.000 |
| Barthel index score | 3.473 | 3.691 | 0.121 | 0.941 | 0.03 |
| Morse score | -3.863 | 2.380 | -0.116 | -1.623 | 0.011 |

**Table S5**. Results of multivariable linear regression analysis of the quality of life in patients with Wilson’s disease (n=134)

HAMD: Hamilton Depression Rating Scale, HAMA: Hamilton Anxiety Scale, UWDRS: Uniform Wilson Disease Rating Scale, MRI: magnetic resonance imaging

**Competing financial interests:** The authors declare no competing financial interests
